# SARS-CoV-2 Outbreak among Malayan Tigers and Humans, Tennessee, USA, 2020

**DOI:** 10.3201/eid2804.212219

**Published:** 2022-04

**Authors:** Heather N. Grome, Becky Meyer, Erin Read, Martha Buchanan, Andrew Cushing, Kaitlin Sawatzki, Kara J. Levinson, Linda S. Thomas, Zachary Perry, Anna Uehara, Ying Tao, Krista Queen, Suxiang Tong, Ria Ghai, Mary-Margaret Fill, Timothy F. Jones, William Schaffner, John Dunn

**Affiliations:** Centers for Disease Control and Prevention, Atlanta, Georgia, USA (H.N. Grome, A. Uehara, Y. Tao, K. Queen, S. Tong, R. Ghai);; Tennessee Department of Health, Nashville, Tennessee, USA (H.N. Grome, M.-M. Fill, T.F. Jones, J. Dunn);; Knox County Health Department, Knoxville, Tennessee (B. Meyer, E. Read, M. Buchanan);; University of Tennessee College of Veterinary Medicine, Knoxville (A. Cushing);; Cummings School of Veterinary Medicine at Tufts University, North Grafton, Massachusetts, USA (K. Sawatzki);; Tennessee Laboratory Services, Nashville (K.J. Levinson, L.S. Thomas, Z. Perry);; Vanderbilt University School of Medicine, Nashville (W. Schaffner)

**Keywords:** COVID-19, respiratory infections, severe acute respiratory syndrome coronavirus 2, SARS-CoV-2, SARS, coronavirus disease, zoonoses, viruses, coronavirus, Malayan tigers, Tennessee, outbreak investigation, United States

## Abstract

We report an outbreak of severe acute respiratory syndrome coronavirus 2 involving 3 Malayan tigers (*Panthera tigris jacksoni)* at a zoo in Tennessee, USA. Investigation identified naturally occurring tiger-to-tiger transmission; genetic sequence change occurred with viral passage. We provide epidemiologic, environmental, and genomic sequencing data for animal and human infections.

In October 2020, the Tennessee Department of Health (Nashville, Tennessee, USA) was notified of severe acute respiratory syndrome coronavirus 2 (SARS-CoV-2) infection in 3 Malayan tigers (*Panthera tigris jacksoni*) at a zoo in the state. Felids, including domestic cats and exotic big cats, have greater susceptibility to SARS-CoV-2 infection than other species (*1*–*4*). Infected domestic cats can transmit the virus to other cats via respiratory droplets or direct contact (*4**–**6*). However, the risk for cat-to-human transmission remains unclear. We investigated the SARS-CoV-2 outbreak in Tennessee to determine its source and provide recommendations to control the spread of infection.

## The Study

Tiger 1, the index case, began showing clinical signs of coronavirus disease (COVID-19), including lethargy, anorexia, and nonproductive cough, on October 12, 2020. Subsequent onset of clinical signs occurred in tiger 2 on October 16 and in tiger 3 on October 17. Oropharyngeal swab specimens were collected under sedation on October 19 and October 27 for SARS-CoV-2 diagnostic testing and submitted to the Runstadler Laboratory, Cummings School of Veterinary Medicine at Tufts University (North Grafton, Massachusetts, USA) for initial testing for open reading frame (ORF) 1b-nsp14 (*7*). Presumptive positive SARS-CoV-2 diagnoses were made in all 3 animals by real-time reverse transcription PCR (RT-PCR) performed by using Mag-Bind Viral RNA Xpress Kit (Omega BioTek, https://www.omegabiotek.com) and UltraPlex 1-Step ToughMix (Quantabio, https://www.quantabio.com). Tiger specimens were then sent to the US Department of Agriculture National Veterinary Services Laboratories (Ames, Iowa, USA) for confirmation by RT-PCR and whole-genome sequencing (*3*).

We conducted an environmental assessment at the zoo on October 29, 2020. The tiger exhibit has 3 primary outdoor visitor viewing areas: 2 outdoor viewing areas with fencing between humans and animals creating a separation of ≈6 feet, and 1 outdoor overhead viewpoint where visitors could view animals from a platform >8 feet above the tiger enclosure. The tiger’s off-exhibit den areas have concrete walls between each animal enclosure and metal interior caging with open airflow that enables keepers to see the tigers. The den areas are directly adjacent to or across from each other.

We observed consistent use of personal protective equipment by zoo employees and veterinary students according to zoo policy. All employees and students in close contact with the tigers before the animals began to show signs of illness wore disposable gloves and cloth facemasks. After the onset of clinical signs of illness and SARS-CoV-2 testing in tigers, persons in the tiger den area wore protective coveralls, disposable gloves, and plastic face shields. At the time of this outbreak in October 2020, fewer data existed for relative mask efficacy, and mask types were not further specified by zoo policy. 

Before and after onset of animal illness, cleaning practices in the off-exhibit cages included use of high-pressure water hoses to clean the floors. Staff used disinfectants daily in the den area, and we confirmed disinfectants were on the US Environmental Protection Agency’s List N: Disinfectants for Coronavirus (COVID-19) (https://www.epa.gov/coronavirus/about-list-n-disinfectants-coronavirus-covid-19-0). All zoo employees and veterinary students used a self-reported evaluation tool via mobile phone that screened for COVID-19 symptoms before their shifts. Zoo visitors were encouraged to wear masks; masks were not required in outdoor spaces at the time of this outbreak.

We also conducted an epidemiologic investigation on October 29, 2020. Our investigation focused on the timeframe beginning 2 weeks before onset of index tiger clinical signs (starting September 28) until date of investigation (October 29). We identified 18 zoo employees and veterinary students who prepared food for or were in close contact with the tigers during this timeframe. For this investigation, we defined close contact to tigers as being within 6 feet of any tiger at the zoo for any length of time during the observation period (September 28–October 29). We selected these proximity criteria based on the US Centers for Disease Control and Prevention (CDC) definition of close contact defined (*8*); however, SARS-CoV-2 transmission can occur from inhalation of virus in the air >6 feet from an infectious source (*9*,*10*). During the week after the index tiger showed signs of illness, community transmission of SARS-CoV-2 was at a 7-day average of 101 new cases/day in the county where this zoo is located, and the 7-day test positivity rate was 10.2%.

**Figure 1:**
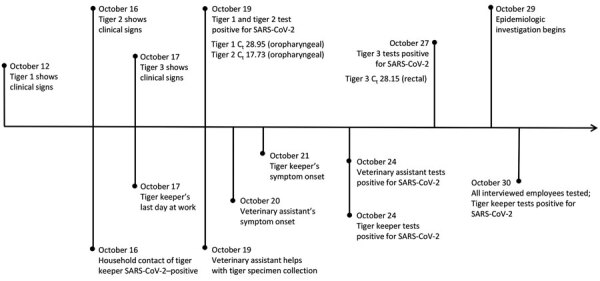
Timeline of events identified during the epidemiologic investigation of an outbreak of SARS-CoV-2 infection among Malayan tigers and humans at a zoo, Tennessee, USA, October 12–30, 2020. Dates related to tiger events are shown above the timeline; dates related to human events are shown below the timeline. C_t_ values for the first positive open reading frame 1b reverse transcription PCR test per animal are shown; methods for extraction and C_t_ value calculation were described previously by Sawatzki et al. (*11*). C_t_, cycle threshold; SARS-CoV-2, severe acute respiratory syndrome coronavirus 2.

We identified 2 employees with COVID-19 during the September 28–October 29 timeframe: a tiger keeper and veterinary clinic assistant. Contact tracing identified an additional household contact to the SARS-CoV-2–positive tiger keeper, but no other SARS-CoV-2–positive contacts were identified. We created a timeline comparing signs of animal illness onset and RT-PCR cycle threshold values with dates of zoo employee symptom onset and testing (Figure 1).

We sent specimens from all zoo employees and veterinary students who tested positive for SARS-CoV-2 to CDC for sequencing and genomic analyses. CDC staff performed whole-genome sequencing as previously described (*12*). We phylogenetically compared tiger sequences with 30 sequences from geographically-associated human SARS-CoV-2 cases collected from the county surrounding the zoo during October 29–November 12, 2020. We also compared tiger sequences with 233 statewide background sequences from specimens collected in Tennessee during March 1–November 12, 2020.

**Figure 2:**
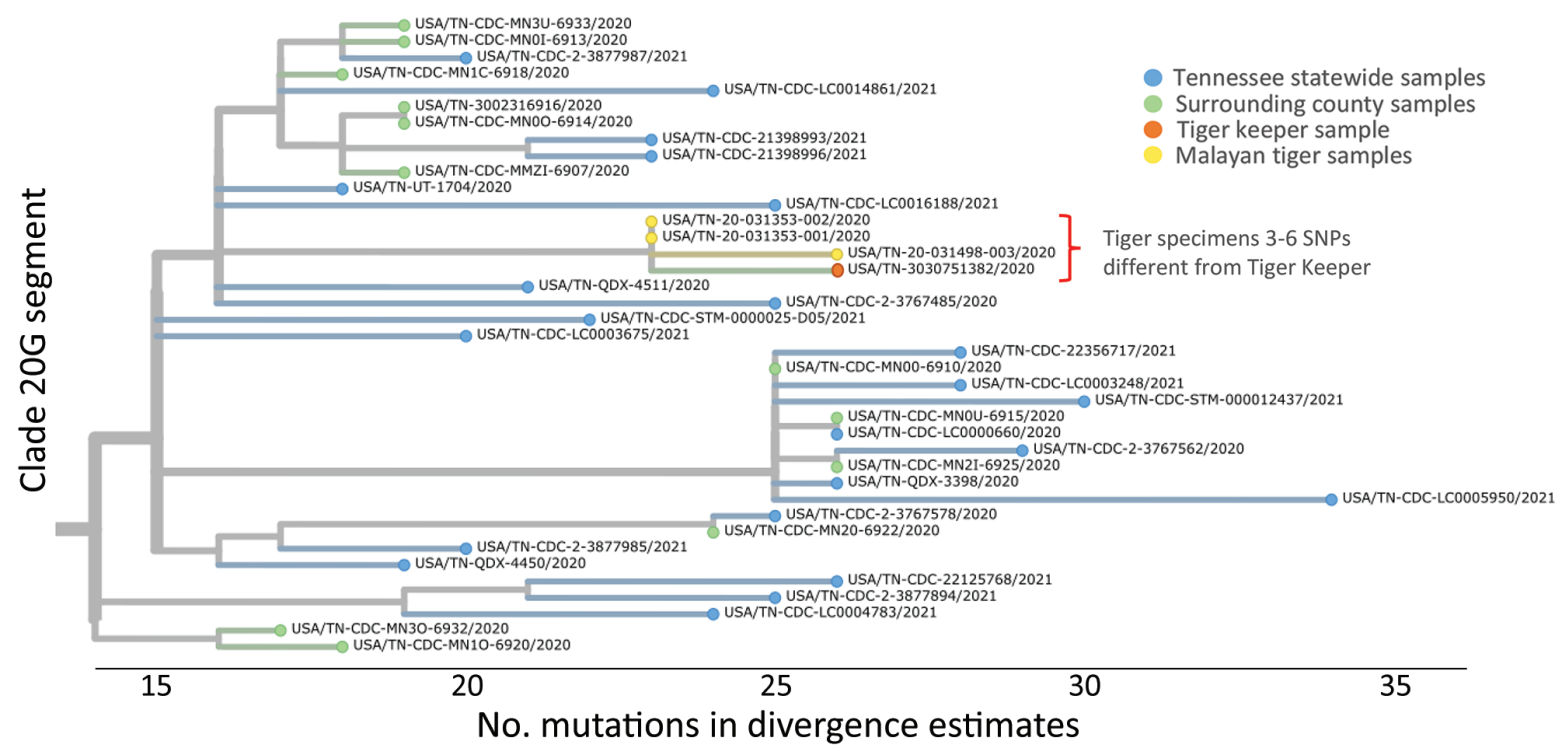
Whole-genome phylogenetic analysis from of an outbreak of SARS-CoV-2 infection among Malayan tigers and humans at a zoo, Tennessee, USA, October 2020. The tree shows a close-up view of clade 20G divergence estimates from the SARS-CoV-2 Wuhan-Hu-1 reference genome and sequences from humans living in Tennessee and Malayan tigers sampled during the outbreak investigation. Sequence analysis showed 3–6 SNP differences between 1 human tiger keeper and all 3 tiger sequences (GISAID accession nos. EPI_ISL_292844–6). Differences are indicated by 1-step edges (lines) between colored dots (individual SARS-CoV-2 sequenced infections). Numbers indicate unique sequences. Phylogenetic relationships were inferred through approximate maximum-likelihood analyses implemented in TreeTime (*13*) by using the NextStrain pipeline (*14*). All high-quality genome sequences from Tennessee were downloaded from the GISAID (https://www.gisaid.org) database on March 16, 2021. Pangolin lineages for investigation sequences were assigned on March 16, 2021. Not all analyzed sequences are shown in this figure because some were outside clade 20G. CDC, Centers for Disease Control and Prevention; SARS-CoV-2, severe acute respiratory syndrome coronavirus 2; SNP, single-nucleotide polymorphism.

Most viral sequences clustered into NextStrain Clade 20G and Pangolin lineage B.1.2, which correspond to the predominant clades observed for human specimens from Tennessee during the time of the outbreak at the zoo. Nucleotide sequence analysis of viral sequences from the tigers (GISAID accession nos. EPI_ISL_2928444–6; https://www.gisaid.org) and the tiger keeper (GenBank accession no. OK170070) also clustered in SARS-CoV-2 clade 20G by Nextstrain tree (Figure 2). The multiple alignment of 4 SARS-CoV-2 genome sequences (tigers 1, 2, and 3 and the tiger keeper) showed a total of 6 single-nucleotide polymorphisms (SNPs) across 4 genomes. Sequences from tigers 1 and 2 were identical and had no substitutions compared with the reference sequence at those 6 SNP positions. Tiger 3’s SARS-CoV-2 sequence contained 3 substitutions that were nonsynonymous G12565T (ORF1a: Q4100H), C17822T (ORF1b: P1452L), and G19889A (ORF1b: R2141K), and the tiger keeper’s SARS-CoV-2 genome sequence had 3 synonymous substitutions (C1498T, C24904T, T26048C) compared with tigers 1 and 2. SARS-CoV-2 genome sequences from the tiger keeper and tiger 3 had 6 SNP differences. Genomic and epidemiologic data suggest that tiger-to-tiger transmission occurred under natural conditions, and genetic change occurred in vivo for tiger 3’s sequence.

## Conclusions

We describe SARS-CoV-2 infection in captive tigers with respiratory clinical signs and provide additional evidence for nonhuman species as hosts for SARS-CoV-2. Findings of this study support tigers’ susceptibility to the virus and potential for sustained transmission among large cats and a risk for zoonotic transmission to humans. The SARS-CoV-2 sequence from the tiger keeper was 3 SNPs different from tigers 1 and 2 and 6 SNPs different from tiger 3. The close genetic relationship between viruses of the tigers and tiger keeper is consistent with the timing of clinical signs of illness and job duties of the tiger keeper, although transmission source or zoonotic transmission cannot be proven from these data alone.

These findings have implications for both the public health and zoologic communities. Zoos should be aware of the possibility of animal infection through incidental exposure by the public or asymptomatic staff members. Humans with known or suspected infection should avoid direct or indirect exposure to susceptible species unless completely unavoidable to avoid potential transmission. Results of this investigation should also prompt zoo and wildlife organizations to reevaluate biosecurity and administrative protocols to minimize risk to and from employees, students, volunteers, and the visiting public interacting with susceptible species.
